# The Diagnosis of Vertebrobasilar Insufficiency Using Transcranial Doppler Ultrasound

**DOI:** 10.1155/2012/894913

**Published:** 2012-11-08

**Authors:** Ibrahim Alnaami, Muzaffer Siddiqui, Maher Saqqur

**Affiliations:** ^1^Division of Neurosurgery, Department of Surgery, University of Alberta, Edmonton, AB, Canada T6G 2B7; ^2^Division of Neurosurgery, Department of Surgery, King Khalid University, P.O. Box 641, Abha, Saudi Arabia; ^3^Division of Neurology, Department of Medicine, University of Alberta, Edmonton, AB, Canada T6G 2B7

## Abstract

*Background*. Vertebrobasilar insufficiency (VBI) is a hemodynamic posterior circulation transient ischemic attack (TIA) caused by intermittent vertebral artery occlusion that is induced by a head rotation or extension. VBI may result from large vessel atherosclerotic disease, dissection, cervical compressive lesions, and subclavian steal phenomenon. Diagnostic transcranial Doppler (TCD) of VBI disease and hemodynamic posterior circulation TCD monitoring in symptomatic positions might prove a useful tool in establishing the diagnosis. *Patient and Material/Method*. A 50-year-old Caucasian man presented with a one-year history of episodic positional vertigo and ataxic gait that were induced by a neck extension and resolved by an upright position or a neck flexion. Computed tomography angiogram (CTA) and TCD confirmed the presence of VBI where no blood flow was detected through posterior cerebral arteries in the symptomatic position (head extension position). *Conclusion*. TCD is a promising noninvasive technique that might have a role as a diagnostic test in VBI.

## 1. Introduction

Vertebrobasilar insufficiency (VBI) is a rare hemodynamic posterior circulation TIA that is caused by intermittent vertebral artery occlusion induced by head rotation or extension [1]. Posterior circulation strokes in general represent 20–30% of all intracranial stokes [2, 3]. In older studies, the stroke rate is the range of 22–35% over five years [4]. Although the term “stroke” is used throughout the literature to refer to the condition, VBI encompasses a wide spectrum of rotational hemodynamic insufficiency that might result from degenerative cervical spine changes [5] and cervical manipulation [6]. VBI may range from a TIA to a frank stroke. The neurological symptoms seldom appear when the contralateral vertebral artery (VA) flow or the collateral flow to the distal vertebrobasilar system from the anterior circulation are sufficient. As VBI is a clinical syndrome, the constellation of vertigo, diplopia, dysarthria, dizziness (brain stem symptoms), and visual defect (occipital cortex symptoms), all or one should raise index of suspicion for VBI [7, 8]. The treatment of VBI ranges from conservative warnings to minimize head movement to surgical procedures designed to limit head rotation or to decompress and free the vertebral artery at the point of compression. 

Different tests can be used in the diagnosis of VBI including magnetic resonance imaging (MRA) [7, 9], single photon emission computed tomography (SPECT) [10], and digital subtraction cerebral angiography (DSA) which is considered the gold standard diagnostic test [11, 12]. TCD is a noninvasive, cheap portable bedside test that allowed for noninvasive evaluation of cerebral hemodynamics within the circle of Willis, vertebral, and basilar arteries. 

We present a unique case of VBI where TCD monitoring plays a role in confirming the mechanism of TIA as being hypoperfusion mechanism rather than embolic phenomenon. 

## 2. Case Report

A 50-year-oldCaucasian man presented with a one-year history of episodic positional vertigo and ataxic gait induced by neck extension and resolved by upright position or neck flexion. He denies any other neurological complains. His general and neurological exams were completely normal. Unterberger's test was negative.

**Figure 1:**
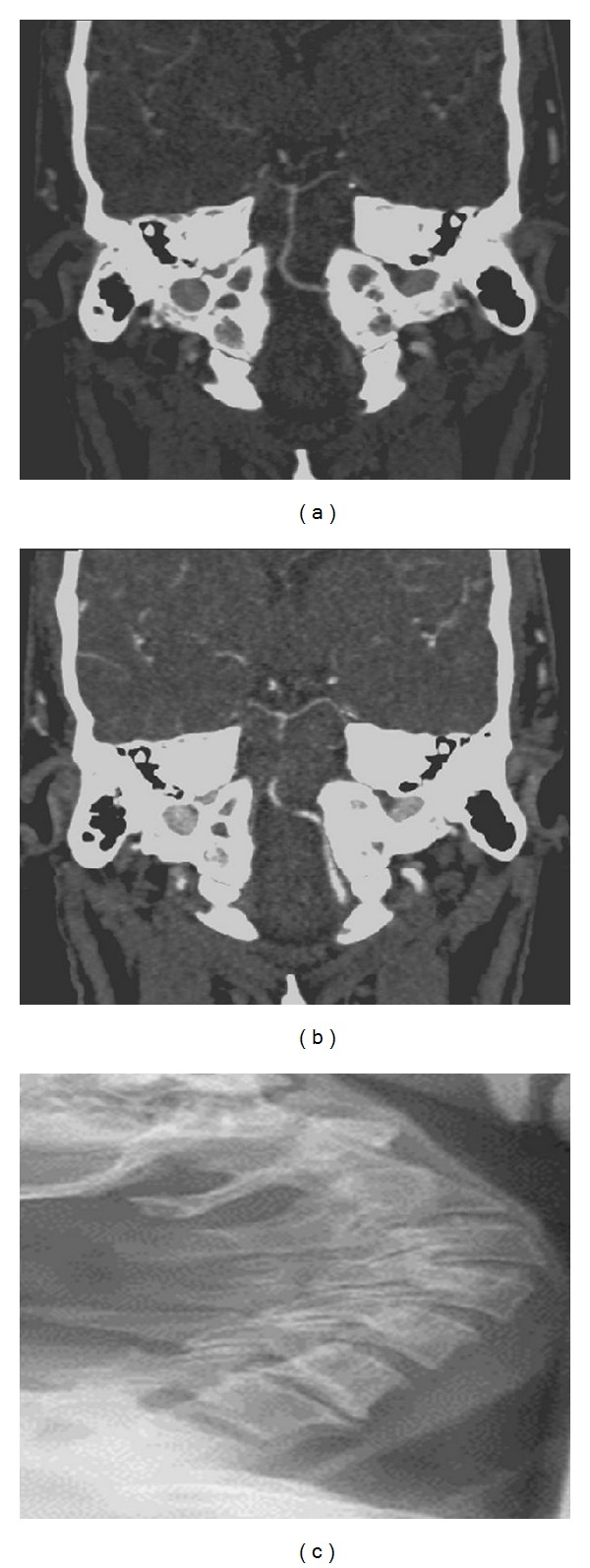
(a) and (b) CTA indicates dominant left vertebral artery and hypoplastic right vertebral artery with minimal contribution in formation of the small basilar artery. (c) Lateral cervical spine X-ray in extension showing diffused degenerative changes with no evidence of cervical instability.

His head computerized tomography (CT) and magnetic resonance imaging (MRI) were within normal limit. His CT angiography of the cerebral vessels revealed a hypoplastic right VA with normal flow in the left VA and BA (Figures 1(a) and 1(b)).

Cervical spine X-ray revealed exaggerated cervical lordosis and diffused degenerative changes with multiple osteophytes at several levels; these changes are worse on cervical 5 and 6 and cervical 6 and 7 junctions (Figure 1(c)).

**Figure 2:**
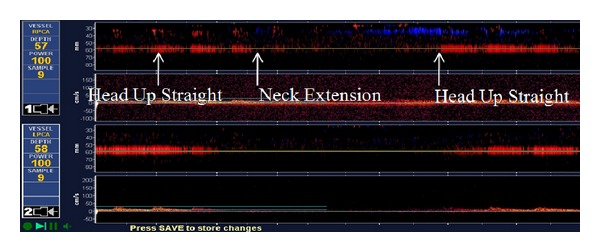
TCD monitoring of bilateral PCAs: bilateral PCA MFV dropped from 19 to 0 cm/sec in the neck extension position.

TCD was performed and did not reveal any intracranial stenosis or occlusion in the anterior or posterior circulation, then 1 hour TCD emboli monitoring of the posterior circulation was performed and was negative for any emboli signal. Finally, TCD monitoring of the bilateral PCA mean flow velocities was monitored in different head position using a head frame to avoid any changes in the angle of insonation (Figure 2). The transforaminal window insonation occurred via the foramen magnum and was first performed at 75 mm depth to locate the terminal vertebral artery (VA) and proximal basilar artery (BA). Insonation of the BA was performed next along its course (range from 80 to 100 mm depth), followed by assessment of the more proximal left and right VAs at depths from 50 to 80 mm by lateral probe positioning. Then the PCAs were insonated through the temporal window by posterior angulation of the probe at depth of 55–75 mm. TCD revealed the followings: the mean flow velocity (MFV) on left posterior cerebral artery (PCA) and right PCA in upright position is the 18 cm/sec and 19 cm/sec, respectively. Then the MFV dropped to 0 cm/sec in the symptomatic position (neck extension) in both vessels. Then, the unaffected right middle cerebral artery (MCA) MFV blood flow was monitored simultaneously with the PCA flow for comparison in different head positions. It revealed the following: the right MCA MFV was 47 cm/sec in upright position and 48 cm/sec in the symptomatic position (neck extension) with no major drop in the MFV. While doing the neck maneuver, the patient became clinically symptomatic (Figure 2). As part of workup, an ear, nose, and throat (ENT) consultation was placed and the patient received a full inner ear examination and workup was negative. Electronystagmography (ENG) was negative. MRI cervical spine rolled out myelopathy. The patient was treated conservatively after he denies spine surgery consultation.

## 3. Discussion

 In our case, we demonstrate a unique role of TCD monitoring in defining the mechanism of hypoperfusion syndrome in posterior circulation TIA by demonstrating a clear drop in the blood flow velocities of the symptomatic vessel during the clinically symptomatic position. 

 VBI diagnosis starts by bedside tests that can elicit the symptoms and solidify the suspicion. Maigne's and Hauten's tests and vertebral artery bruit are helpful bedside tests in patients with suspicious VBI. Several neuroimaging modalities have been used to evaluate the CBF of the brain in patients with hypoperfusion TIAs. Xenon-133 inhalation, xenon-enhanced CT scanning, SPECT scan, and positron emission tomography with fluorodeoxyglucose have been used to determine the regional CBF [13]. However, dynamic cerebral angiography (DCA) is considered the gold standard procedure in VBI diagnosis [11, 12].

TCD is a noninvasive and inexpensive monitoring tool that can be performed at bedside to assess the hemodynamic status of the cerebral vessels in different body positions as we demonstrated in our case. The TCD monitoring of the affected vessel demonstrated the drop in blood flow during the symptomatic head position rather than being an embolic phenomenon in the presence of critical arterial stenosis. This piece of information is critical in planning further management and planning a preventive measure like avoidance of cervical manipulation [14].

Schneider et al. in 1991 published a series in which they started the trend of using TCD as a noninvasive procedure in VBI diagnosis [15]. It was followed by other series by Cher et al. in 1992 where they compared the results of TCD with DSA in 20 patients with VBI. They found that TCD had a sensitivity of 87%, a specificity of 80%, a positive predictive value of 93%, and a negative predictive value of 67%. They concluded with saying that TCD is a useful screening method in patients with VBI to detect large vessel disease of intracranial vertebrobasilar system and had a limited benefit to diagnose proximal vertebral artery stenosis [16]. Despite the excellent TCD evidence of compromised flow in PCA's in symptomatic position, it does not always imply inadequate blood flow in more proximal basilar or vertebral arteries, as the latter might get sufficient flow from collaterals like ascending cervical artery and others.

VBI remains a clinical syndrome that can be mimicked by inner ear diseases, cervical myelopathy, and other. Adjuncts to guide in making diagnosis and treatment are required. Our case demonstrates a new role of hemodynamic TCD monitoring, by establishing the clear drop in blood flow during the symptomatic position. This might help establishing the diagnosis of VBI syndrome and guide a different type of conservative and surgical treatment. Further studies are needed to answer this question in this rare phenomenon.

## 4. Conclusion

VBI remains a challenging disease to diagnose due to lack of hemodynamic monitoring during the symptoms. TCD is a promising noninvasive technique that might have role as a diagnostic test in VBI.
